# Multiomics Analysis Reveals Aberrant Metabolism and Immunity Linked Gut Microbiota with Insomnia

**DOI:** 10.1128/spectrum.00998-22

**Published:** 2022-10-03

**Authors:** Qinghua Wang, Bin Chen, Dashuang Sheng, Junjie Yang, Shujie Fu, Jingwen Wang, Changying Zhao, Yihui Wang, Xiangzhen Gai, Jianfeng Wang, Kyle Stirling, Xueyuan Heng, Honghao Man, Lei Zhang

**Affiliations:** a College of Life Science, Shandong Normal University, Jinan, China; b School of Biological Science and Technology, University of Jinan, Jinan, China; c Department of Biostatistics, School of Public Health, Cheeloo College of Medicine, Shandong University, Jinan, China; d Microbiome-X, National Institute of Health Data Science of China & Institute for Medical Dataology, Cheeloo College of Medicine, Shandong University, Jinan, China; e College of Life Science, Qilu Normal University, Jinan, China; f Department of Neurology, Weihai Central Hospital Affiliated to Qingdao University, Weihai, China; g Shandong Children’s Microbiome Center, Qilu Children's Hospital, Cheeloo College of Medicine, Shandong University, Jinan, China; h Luddy School of Informatics, Computing and Engineering, Indiana University, Bloomington, Indiana, USA; i Department of Neurosurgery, Lin Yi People’s Hospital, Linyi, China; Wayne State University

**Keywords:** gut microbiota, gut-brain axis, immune system, insomnia, metabolomics

## Abstract

Studies have confirmed that insomnia is related to gut microbiota. Previous research suggests that immunity and metabolism are also associated with insomnia. However, to our knowledge, the integration of these factors has not been investigated in insomnia. Here, we explored the correlations across gut microbiota, serum metabolism, and inflammatory factors in insomnia. Our results showed that the composition and structure of gut microbiota and metabolism in insomnia patients were different from healthy controls. Compared to healthy controls, the relative abundances of *Lactobacillus*, Streptococcus, and Lactobacillus crispatus were significantly increased in insomniacs. There were five metabolic pathways in insomniacs (glycerophospholipid metabolism; glutathione metabolism; nitrogen metabolism; alanine, aspartate, and glutamate metabolism; aminoacyl-tRNA biosynthesis) significantly different between the two groups. Moreover, we found that IL-1β levels were significantly higher in insomnia patients while TNF-α was significantly reduced. We further identified that the changes in the level of IL-1β and TNF-α were associated with some specific bacteria and metabolites, such as Prevotella amnii, Prevotella buccalis, Prevotella timonensis, and Prevotella colorans. Mediation analysis further determined that the immune factors and metabolites could mediate the relationship between gut microbes and insomnia.

**IMPORTANCE** Our study indicated that systematic inflammation and metabolites might be a pathway linking the gut microbiome with insomnia. These findings provide new insights and a better understanding of gut microbiota's role in insomnia as well as potential novel microbiome-related etiologies for insomnia.

## INTRODUCTION

Insomnia is the most common sleep disorder among adults with undefined pathogenesis (1), and growing evidence suggests that gut microbiota might play a role in its pathogenesis (2–4). Previous studies have suggested that the incidence of insomnia is linked to biological rhythms, immune function, and nutrient metabolism (5–7). Furthermore, there is considerable evidence showing that the gut microbiome not only affects the digestive, metabolic, and immune functions of the host but also regulates host sleep through the microbiome-gut-brain axis (5, 8). Several studies have provided preliminary evidence for the involvement of gut microbiota in sleep disorders, including in animal models and human studies. For example, in the rodent model, drug-induced insomnia and sleep fragmentation can alter the gut microbiota (9–11). Moreover, altered gut microbiota was found in the feces of patients with clinical insomnia (4, 12, 13). In addition to the composition of the gut microbiota, both signaling pathways and metabolic functions were also perturbed in patients with insomnia disorder (7, 13).

Recently, some researchers have suggested that systematic inflammation and metabolites may be a pathway linking the gut microbiome with insomnia. Patients with acute and chronic insomnia have increased inflammatory cytokines compared to healthy subjects, and insomnia-related signature bacteria showed a correlation with plasma interleukin (IL)-1β (12). Furthermore, the total microbiome diversity and Bacteroidetes and Firmicutes were positively correlated with sleep efficiency and IL-6 concentrations (4). In another study, researchers found that chronic sleep disruption altered gut microbiota and inflammatory factors, inducing systemic inflammation in mice (9). Studies also suggested that poor or decreased sleep caused metabolic changes in peripheral metabolism (14–16).

However, almost no study has used the integrated method to explore the interrelationships among gut microbiota, serum metabolome, and inflammatory cytokines in insomnia. In this study, we analyzed the gut microbiota, serum metabolome, and inflammatory cytokines of insomniacs and compared them with those of control participants presenting with a good sleep history. By combining 16S rRNA gene amplicon-based sequencing, enzyme-linked immunosorbent assays (ELISAs) of inflammatory factors, and untargeted metabolomics, our work revealed a link between host metabolism, immunity, and the microbiome.

## RESULTS

### Distinct gut microbiome observed in insomniacs.

**FIG 1:**
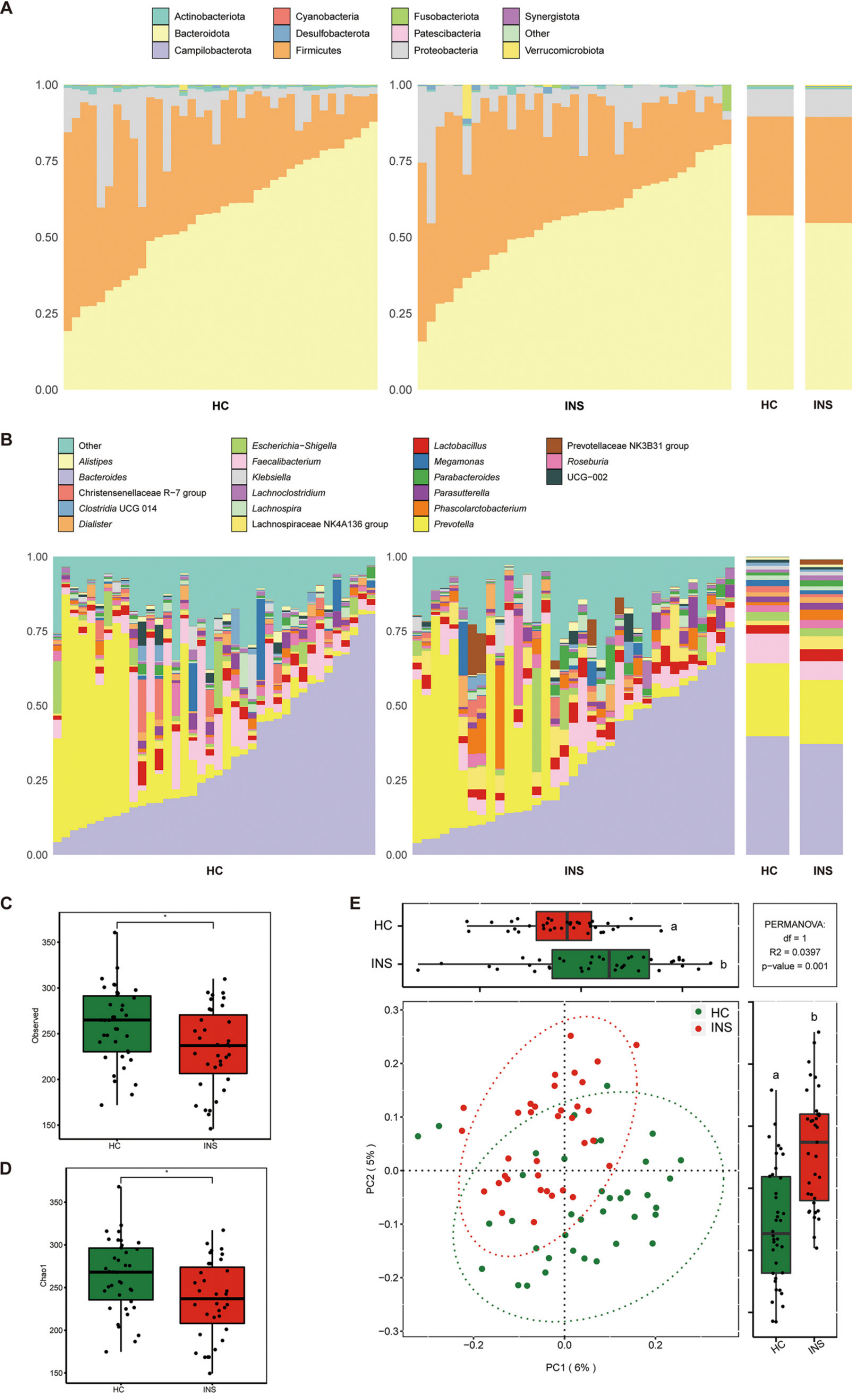
Distinct gut microbiomes were observed in insomniacs (INS) compared to healthy controls (HC). (A and B) Microbiome community structure at the phylum (A) and genus (B) levels compared in INS and HC. (C and D) Observed ASVs (C) and Chao1 index (D) of gut microbiota compared between INS and HC. (E) PCoA and boxplot are shown along the first two principal coordinates of Bray-Curtis distances for INS and HC. Ellipses represent the 95% confidence interval around the group centroid. The *P* value was calculated by PERMANOVA.

**FIG 2:**
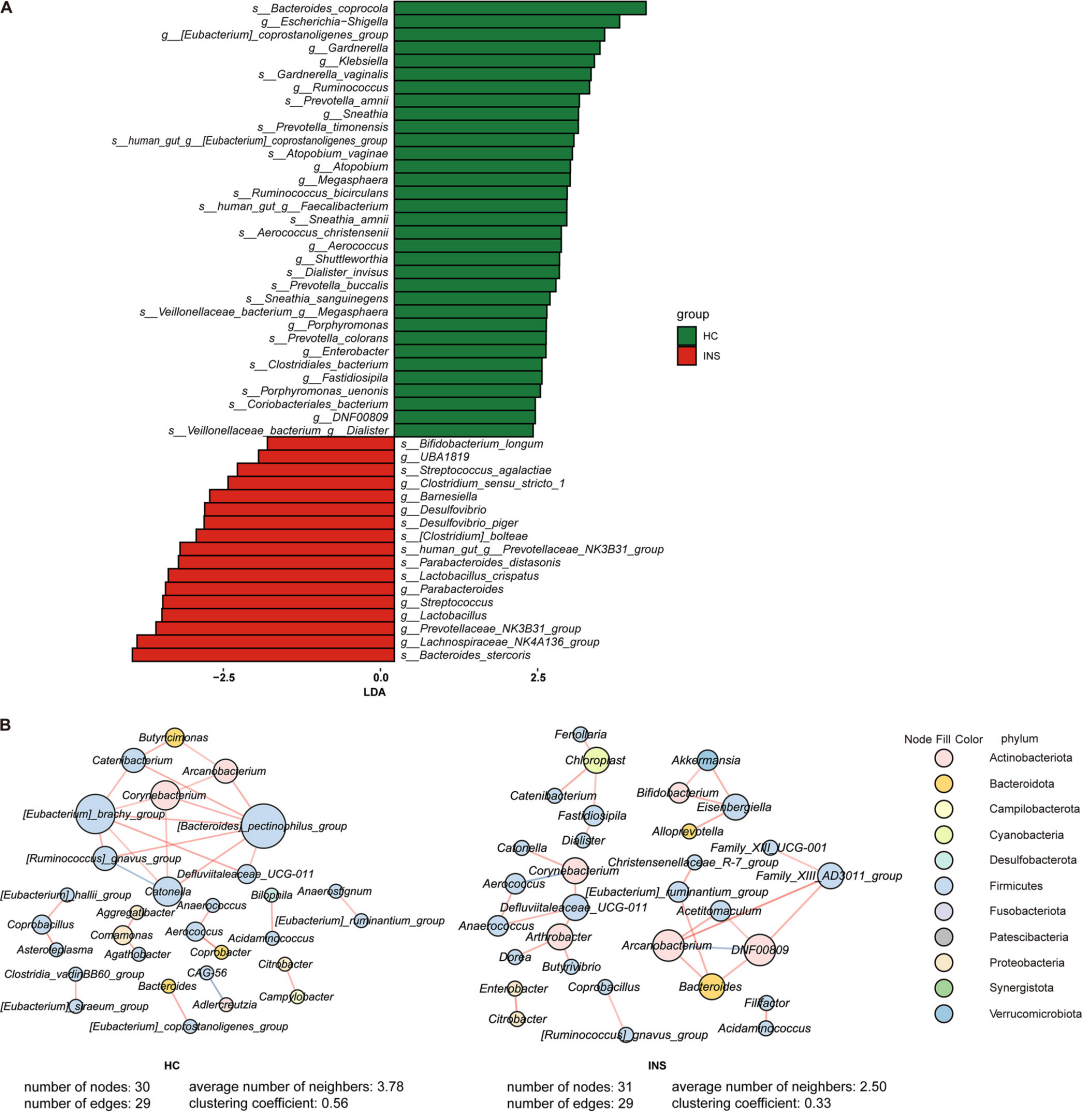
Characteristics of gut microbial community composition in insomniacs (INS) and healthy controls (HC). (A) Significantly different taxonomic biomarkers between INS and HC were identified by LEfSe. (B) The co-occurrence networks in the microbial communities for INS and HC. The red edges indicate positive correlations between two nodes, and the blue edges indicate negative correlations. The network parameters, including the number of nodes, number of edges, average number of neighbors, and clustering coefficient are presented in the figure.

**FIG 3:**
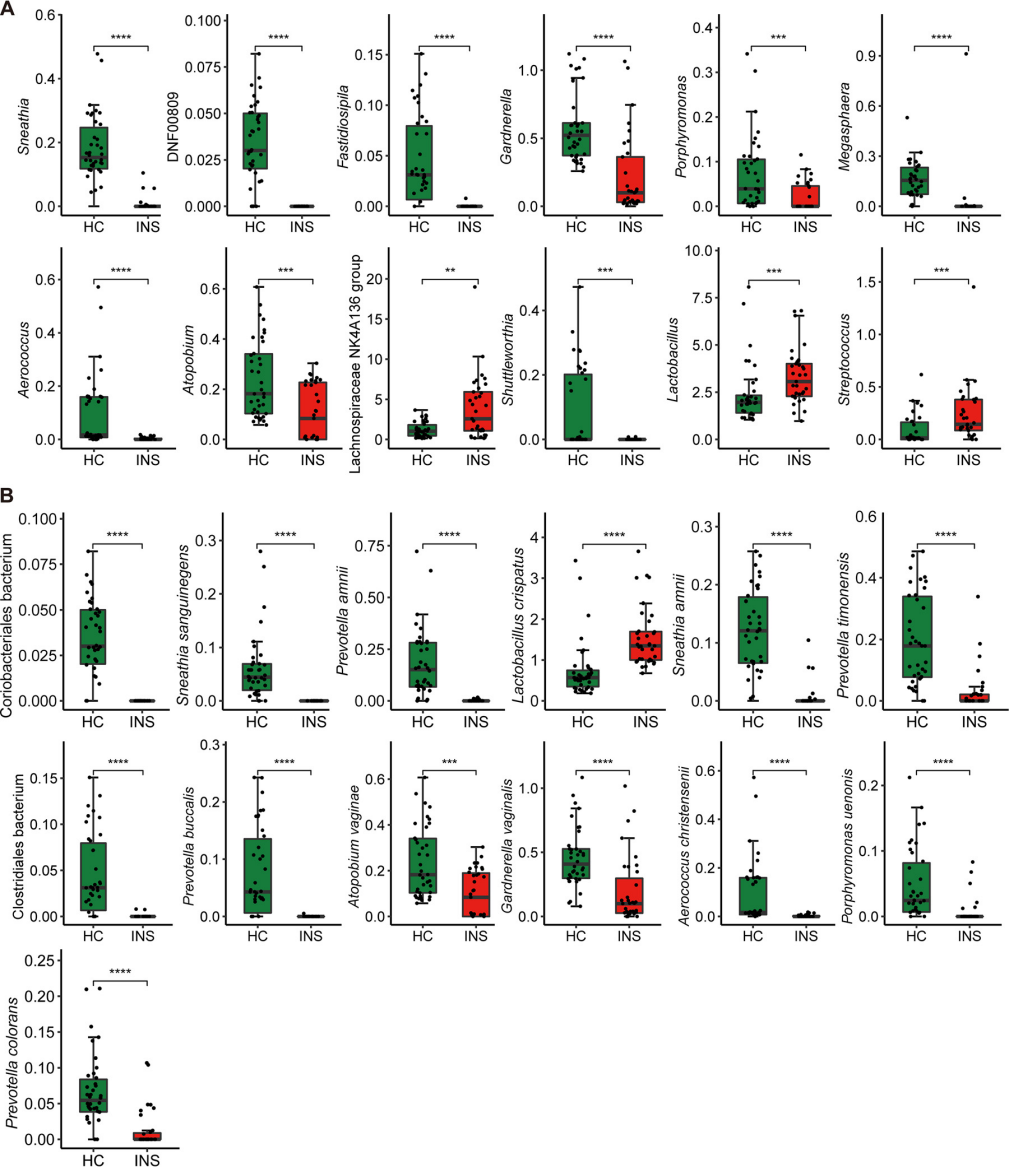
Boxplot of signature microbes in insomniacs (INS) and healthy controls (HC). (A) Genus level. (B) Species level. ***, *P* < 0.001; ****, *P* < 0.0001.

The fecal microbiota composition profiles were analyzed by 16S rRNA gene sequencing. There was variation in the composition of each sample at the phylum and genus levels. At the phylum level (Fig. 1A), the relative abundance of Bacteroidetes in the gut microbiota of insomniacs and healthy controls was 54.69% ± 16.75% and 57.17% ± 19.33%, respectively. This was followed by Firmicutes, which accounted for 33.64% ± 12.93% in insomniacs and 32.02% ± 18.05% in healthy controls, and Proteobacteria, which accounted for 10.62% ± 12.14% and 8.43% ± 9.98% in insomnia patients and healthy controls, respectively. At the genus level (Fig. 1B), the relative abundance of *Bacteroides* in the gut microbiota of insomniacs and healthy controls was 31.36% ± 21.71% and 33.54% ± 22.60%, respectively, followed by *Prevotella*, the relative abundance of which was 18.07% ± 24.35% and 20.63% ± 26.28% in the gut microbiota of the patients and controls, respectively. For alpha diversity, health controls (HC) showed a significantly higher Chao1 index and observed amplicon sequence variants (ASVs) than insomniacs (INS) (Fig. 1C and D), while Shannon and Simpson's index did not differ between the two groups (Fig. S1 in Supplemental File 1). Principal coordinate analysis (PCoA) was used to compare bacterial community patterns between the two groups. The results (Fig. 1E) showed that there was a significant difference in community patterns between insomniacs and healthy controls based on the Bray-Curtis distance (R^2^ = 0.0397, *P* = 0.001). Linear discriminant effect size analysis (LEfSe) was performed with the purpose of screening potential gut microbiota biomarkers related to insomnia. A total of 50 biomarkers in genus and species levels were identified (Fig. 2A). At the same time, 18 genera and 17 species were selected as microbial features by random forest (Fig. S2 in Supplemental File 1). Finally, 12 genera and 13 species in the intersections of LEfSe and random forest were identified as signature microbes, among which the abundance of Lachnospiraceae NK4A136 group, *Lactobacillus*, Streptococcus, and Lactobacillus crispatus were significantly enriched in the gut microbiome of INS compared to that of HC (Fig. 3A and B).

Owing to the significant differences in microbial community structure between INS and HC, we further evaluated the microbial co-occurrence network for each group (Fig. 2B). The results showed that the microbial networks in INS were sparser than in HC. [*Bacteroides*] *pectinophilus* group and [*Eubacterium*] *brachy* group were identified as the top two keystone taxa in microbial communities of HC, while *Arcanobacterium* and DNF00809 (Coriobacteriales bacterium) were in communities of INS. Furthermore, DNF00809, Aerococcus, and *Fastidiosipila* in the co-occurrence network of insomnia were representative genera for HC (Fig. 2B). Altogether, microbiota profiles were significantly different between INS and HC groups.

### Variation of serum inflammatory factors in insomniacs.

We measured serum inflammatory factors, including interleukin-1β (IL-1β), tumor necrosis factor (TNF)-α, and interleukin (IL)-6 using ELISA. The IL-1β was significantly elevated (2.315 ± 2.202 pg/mL in INS versus 0.967 ± 0.745 pg/mL in HC, *P* < 0.0001). Meanwhile, TNF-α was significantly decreased in INS (2.055 ± 1.619 pg/mL in insomniacs versus 3.234 ± 1.520 pg/mL in HC, *P* < 0.0001). However, the IL-6 level was consistent between the INS and HC (1.842 ± 1.396 pg/mL in INS versus 1.805 ± 1.541 pg/mL in HC, *P* = 0.1547) (Fig. S3 in Supplemental File 1).

### Significant alteration of metabolomic profiles in insomniacs.

**FIG 4:**
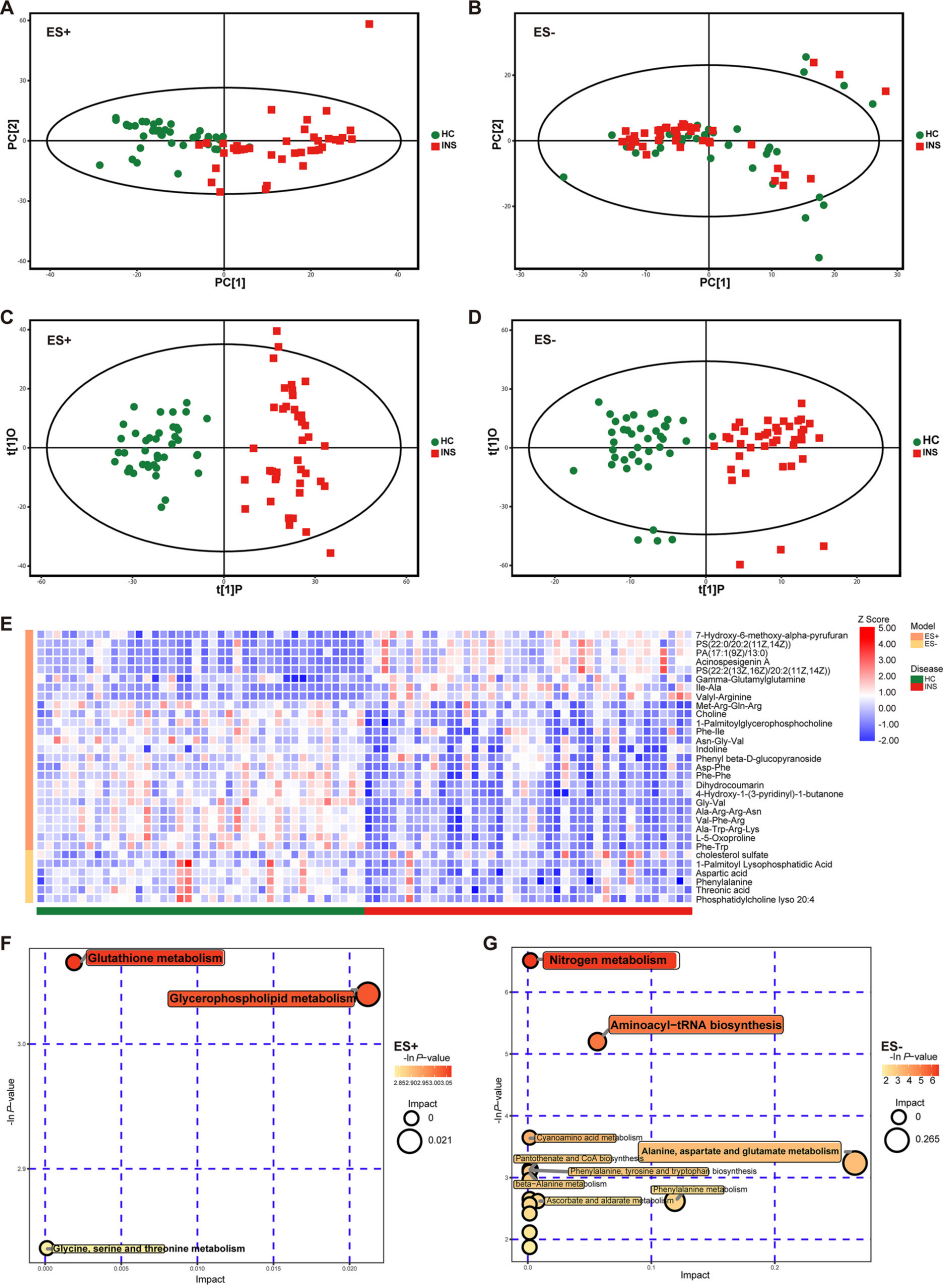
Different metabolites between insomniacs (INS) and healthy controls (HC). (A) Score scatterplot of PCA model (ES+). (B) Score scatterplot of PCA model (ES−). (C) Score scatterplot of OPLS-DA model (ES+). (D) Score scatterplot of OPLS-DA model (ES−). (E) A heatmap illustrating that the relative amounts of metabolites varied in insomniacs and healthy controls. Data were transformed into Z scores in the heatmap. (F and G) KEGG pathways were determined by functional enrichment analysis of differential ES+ (F) and ES− (G) metabolites.

Metabolic profiling of the INS and HC were acquired by Ultra performance liquid chromatography-tandem mass spectrometer (UHPLC-MS/MS). In total, 5,831 peaks were detected, and 5,736 peaks remained after using a relative standard deviation denoising method in positive ion mode (ES+). In negative ion mode (ES−), 2,612 peaks were detected, and 2,588 peaks remained after denoising. To discriminate the metabolic profiles between INS and HC, we used PCA and orthogonal partial least-squares discriminant analysis (OPLS-DA). The results indicated a large separation of the metabolome between insomniacs and healthy controls in PCA plots (Fig. 4A and B) both in ES+ (R^2^X = 0.32) and ES− (R^2^X = 0.317). Furthermore, individuals in the insomniac group were separated from those in healthy controls as further evidenced by the OPLS-DA score scatterplots (Fig. 4C and D) both in ES+ (R^2^X = 0.169, R^2^Y = 0.905, Q^2^ = 0.707) and ES− (R^2^X = 0.175, R^2^Y = 0.84, Q^2^ = 0.532). We performed a permutation test to further validate the OPLS-DA model. After 200 permutations, the R^2^ intercept was 0.71 and 0.78 in ES+ and ES−, respectively, and the Q^2^ intercept values were −0.87 and −0.73 in ES+ and ES−, respectively (Fig. S4A and B in Supplemental File 1). To screen for differential metabolites, the first principal component of variable importance in the projection (VIP) was obtained (Fig. S4C and D in Supplemental File 1). The VIP values exceeding 1 were first selected as differential metabolites, and the Student's *t* test also was used. We found 25 metabolites in ES+ and 6 metabolites in ES− (VIP > 1, *P* < 0.05; Table S2 in Supplemental File 1), which was visualized with a heatmap (Fig. 4E). We found that, in ES+, 1-palmitoylglycerophosphocholine was significantly increased. However, 7-hydroxy-6-methoxy-alpha-pyrufuran, acinospesigenin A, PS(22:2[13Z,16Z]/20:2[11Z,14Z]) and PA(17:1[9Z]/13:0) were significantly decreased in the serum of INS. Meanwhile, aspartic acid, phenylalanine, and phosphatidylcholine lyso 20:4 in ES− were significantly decreased in INS. All differential metabolites were then subjected to regulatory pathways analysis to discover the metabolic pathways exhibiting high correlations with the metabolites. According to *P* value and influence value, significant abnormalities were found in five metabolic pathways in insomniacs: glycerophospholipid metabolism (*P* < 0.05); glutathione metabolism (*P* < 0.05); nitrogen metabolism (*P* < 0.01); alanine, aspartate and glutamate metabolism (*P* < 0.05); aminoacyl-tRNA biosynthesis (*P* < 0.05) (Fig. 4F and G).

### Inflammatory factors and metabolites were associated with gut microbiota.

**FIG 5:**
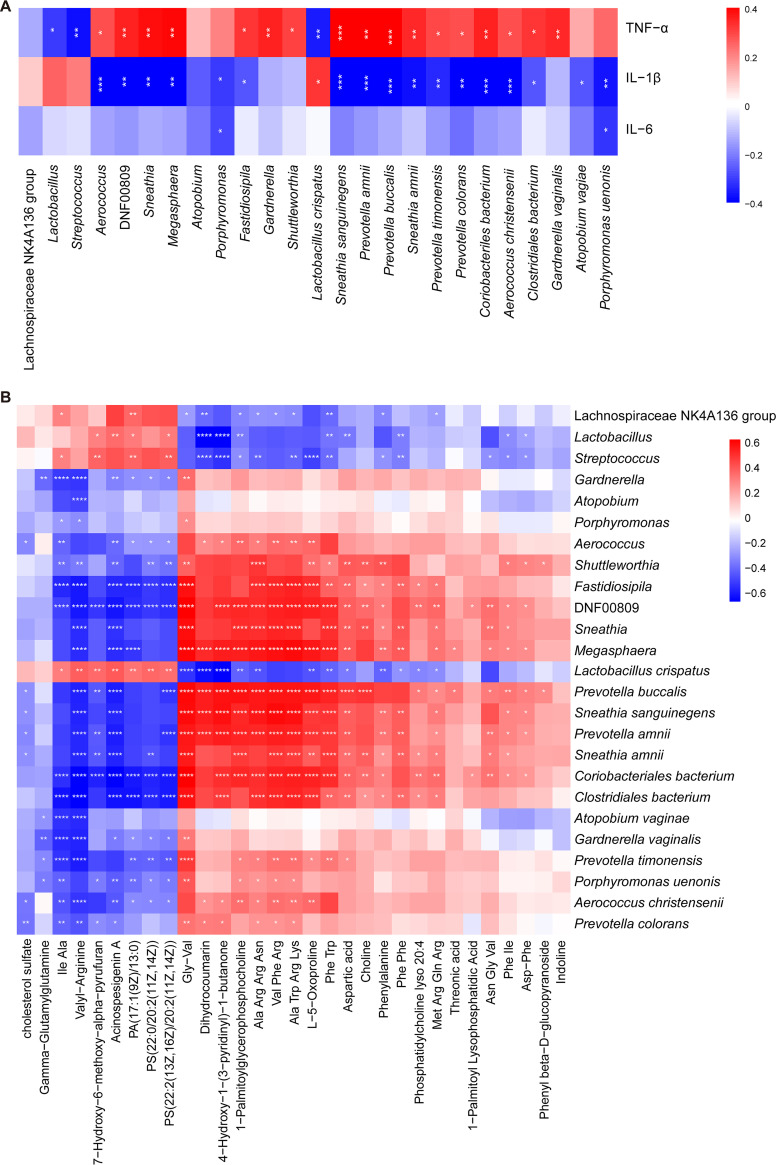
Correlation of gut microbiota with inflammatory factors and serum metabolites. (A) Heatmap of Spearman correlation analysis between gut microbiota and inflammatory factors. (B) Heatmap of Spearman correlation analysis between the gut microbiota and metabolites. Red and blue indicate positive and negative correlations, respectively. *, *P* < 0.05; **, *P* < 0.01; **, *P* < 0.001.

To understand the potential correlation between the altered gut microbiota and the inflammatory factors in the insomniacs, we performed a Spearman correlation analysis (Fig. 5A). Most genera and species were positively correlated with TNF-α, while only *Lactobacillus* and Streptococcus were negatively correlated with TNF-α. Interestingly, these two genera significantly increased in INS (Fig. 3). However, IL-1β was significantly negatively correlated with most genera and species, and only *Lactobacillus* was positively correlated with it. At the same time, we noted a significant correlation between Prevotella amnii, Prevotella buccalis, Prevotella timonensis, Prevotella colorans, and TNF-α/IL-1β.

The correlations between the varied metabolites and the gut microbiota were identified (Fig. 5B). Most of the metabolites that were significantly increased in INS were positively correlated with gut bacteria significantly increased in INS and negatively correlated with gut bacteria significantly decreased in INS, and vice versa. It indicated that this metabolic level variation might be partially due to differences in gut microbiota. We found that many amino acids and short peptides were reduced in the serum of patients with insomnia, such as aspartic acid, phenylalanine, Phe-Phe, Asp-Phe, Phe-Trp, etc. The four bacteria of the genus *Prevotella* that were significantly reduced in INS were positively correlated with most of them, suggesting that *Prevotella* may play a protective role by promoting the production of related amino acids in the host.

**FIG 6:**
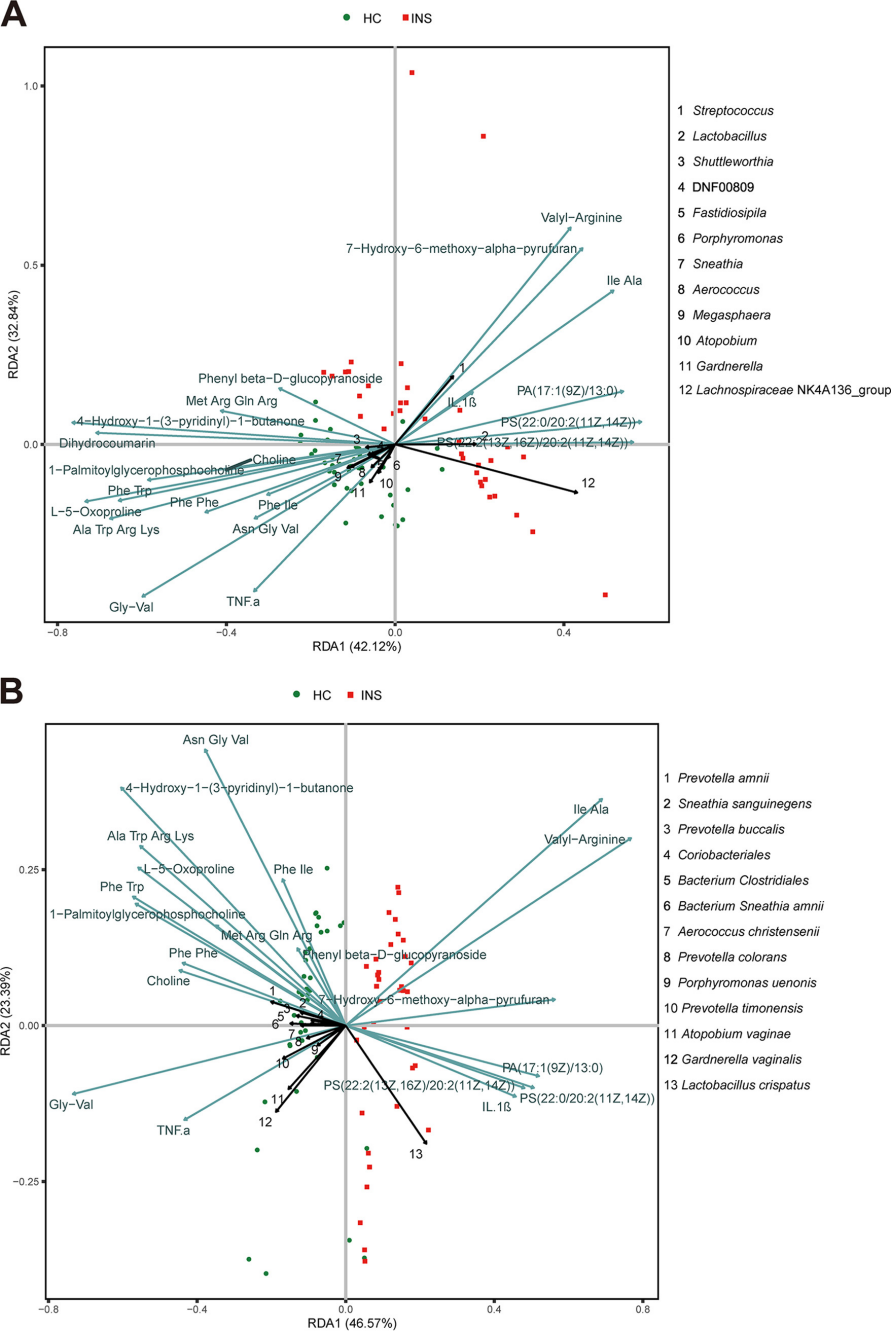
Redundancy analysis (RDA) between immune factors/differential metabolites and signature microbes. (A) Genus level. (B) Species level. Green and black segments indicate immune factors/differential metabolites and signature microbes, respectively.

Subsequently, we explored the association of immune factors and differential metabolism, termed environment variables, with the differential species and genera using redundancy analysis (RDA). Consistent with the correlations mentioned above, the representative species and genera had a good correlation with the environment variables (Fig. 6). The significantly increased metabolites and immune factors in INS were positively correlated with Streptococcus, Lachnospiraceae NK4A136 group, *Lactobacillus*, and Lactobacillus crispatus, which were signature microbes for INS. The environmental variables accounted for 22.53% and 34.64% of the variation of representative species and genera, respectively. It also showed that these factors could well differentiate the INS and HC.

### Inflammatory factors and metabolites mediated the relationship between gut microbiota and insomnia.

We were interested in whether inflammatory factors or metabolites mediate the relationship between gut microbiota and insomnia. Mediation analysis revealed multiple inflammatory factors and metabolites in serum influencing the relationship between gut microbiota and insomnia. We identified inflammatory factors and metabolites with evidence for strong microbiota-INS effects (Table S3 and Fig. S5 and S6 in Supplemental File 1). *Fastidiosipila*, *Shuttleworthia*, Streptococcus, Prevotella buccalis, *Clostridiales* bacterium, DNF00809, *Megasphaera*, and *Aerococcus* were correlated with insomnia mediated by more than 10 metabolites and inflammatory factors. In all, these results suggested that inflammatory factors and metabolites mediate the effect of gut microbiota on insomnia.

## DISCUSSION

The gut microbiome not only affects the immune functions of the host but also potentially regulates sleep through the microbiome-gut-brain axis (5). However, the study on the effects of gut microbiota on the pathogenesis of insomnia is incomplete. Our study extends the findings of previous studies in several important ways. Liu et al. (13) demonstrated that the composition, diversity, and metabolic function of gut microbiota were significantly changed between insomnia patients and healthy controls. But the assessment of metabolic function is only based on PICRUSt function prediction. Recent studies have also shown abnormalities in the metabolome of insomnia patients (17). Our work further identified abnormalities in serum metabolites associated with gut microbiota in patients with insomnia and identified similarities with previous findings by metabolomic analysis. Here, our study comprehensively integrates gut microbiota, serum metabolites, and inflammatory factors in insomniacs. This information may lead to a better understanding of the bidirectional communication between the host and the gut microbiome and may produce novel strategies for insomnia treatment and intervention.

The immune system plays a crucial part in sleep, especially gut microbiota-mediated inflammation may be a major mechanism of interaction between sleep disturbances and gut microbiota (18). Increased gut microbiota-derived lipopolysaccharide (LPS) contributes to the production of proinflammatory cytokines such as IL-1β, TNF-α, and IL-6, which have been shown to play a key role in sleep disturbances (2, 4, 12). The levels of IL-1β and TNF-α in the serum of the insomniacs were significantly different in our study. Both factors have been reported to affect the brain, leading to decreased appetite, sleep disturbance, and depression (19). Similar to our study, it has also been found that the levels of IL-1β in insomnia were significantly increased compared to healthy subjects (12). Our study enriches relevant evidence and further discovers new associations between gut microbes and immunity in insomnia.

There is growing evidence that metabolites of gut microbiota are important sources of sleep-promoting signals (2). The metabolomic study exhibited that multiple serum metabolites were distinct in the insomnia group, of which some were consistent with reported insomnia markers (17), such as gamma-glutamyl-glutamine, phenylalanine, and phosphatidylcholine lyso. Higher gamma-glutamyl-glutamine levels were associated with later sleep timing (15). We found that many amino acids and short peptides were reduced in the serum of insomniacs. Aspartic acid was an endogenous amino acid with important neuroendocrine effects (20). At the same time, a study has shown that the level of aspartic acid was reduced in the hippocampus of rats with fragmented sleep (21). Phenylalanine and tryptophan were precursors for the synthesis of serotonin and the further synthesis of melatonin (22), which was involved in the regulation of sleep (23). Importantly, we found that Prevotella amnii, Prevotella buccalis, Prevotella timonensis, and Prevotella colorans enriched in HC were negatively correlated with IL-1β levels while positively correlated with amino acids and short peptides, revealing that *Prevotella* plays an important role in the protection of insomnia. It has been reported that *Prevotella* was a proficient producer of short-chain fatty acid propionate (24). Further, we found that amino acids, including Valyl-Arginine, Phe-Phe, Phe-Trp, Ala-Trp-Arg-Lys, and immune factor, IL-1β, could mediate the relationship between species belonging to *Prevotella* and insomnia, indicating that *Prevotella* may affect sleep by regulating amino acid metabolism and promote inflammatory. This information may lead to a better understanding of the bidirectional communication between the host and the gut microbiome and may produce novel strategies for insomnia treatment and intervention.

Although we had a much larger sample size than any of the previous studies about the association between insomnia and gut microbiota, the sample size of our study was still somewhat small. Furthermore, we performed a bioinformatics analysis combined with multiple data types. However, there are some limitations to our current study. First, this is a single-center cohort study that cannot be generalized to other populations. Thus, it led to differences in conclusions compared with other studies. In the second place, there are limitations in the clinical information and demographic characteristics collected. Given these limitations, these data must be confirmed in multicenter clinical trials from different regions of the world.

Our study provides a comprehensive comparison of the differences in gut microbiota, serum metabolites, and serum immune factors between insomniacs and healthy controls. Moreover, we identified key gut bacteria and metabolites that might serve as potential biomarkers related to insomnia. Our findings provided new insights into the interactions among gut microbiota, immunity, and metabolism and explore the role of gut microbiota in the development of insomnia. Our study provided multiomics data for further studies of insomnia and an important foundation for subsequent large sample validation and longitudinal studies.

## MATERIALS AND METHODS

### Study design and participants.

This study was approved by the Institutional Review Board of the Weihai Central Hospital (IRB number WCH2017-1201). Informed consent was obtained from all participants. Procedures were carried out in this study to conform with the ethical standards stipulated by the institutional and national research committee, as well as those stipulated in the 1964 Declaration of Helsinki and its later amendments or comparable ethical standards.

Our study recruited 40 insomnia patients (INS) and 40 healthy control participants with good sleep histories (HC) (Table S1 in Supplemental File 1). The following inclusive criteria were used. Participants met the criteria for primary insomnia defined according to the Diagnostic and Statistical Manual of Mental Disorders (DSM-5) that included (i) unsatisfied with the time or the quality of sleep, including difficulty in falling asleep, prolonged sleep, and early awakening; (ii) suffering from sleep disorders that cause serious daytime dysfunction or damage (such as emotional or cognitive disorders, work dysfunction); (iii) insomnia attacks at least three nights a week and sleep disorders occurring for at least 3 months; (iv) the presence of sleep disorders occurring even though there are adequate sleep opportunities. The healthy control participant volunteers were recruited from the people also receiving a physical examination at the same time. Participant age was limited to those 18 years old or above. The exclusion criteria were established as follows: secondary insomnia caused by somatic or psychiatric diseases; suffering from diabetes, infectious diseases, gastrointestinal diseases, and other diseases; smokers and drinkers; those who could not communicate and cooperate normally; those with a history of radiotherapy and chemotherapy; pregnant women; and anyone that had taken antibiotics in the previous 3 months.

### Sample collection and preparation.

The feces samples were collected by fecal collectors that were preloaded with 600 to 800 mL of absolute ethanol (25). The samples were immediately frozen in a −80°C refrigerator. Three samples were collected from each participant. One was used for the extraction of DNA. The other two samples were reserved for further studies. All the venous blood samples were extracted between 8:30 to 10:00 AM using vacutainer tubes without anticoagulants (serum), and then the samples were stratified at room temperature for 1 h. The supernatant of the blood was saved after 3,000 rpm centrifugation at room temperature for 1 min. The serum samples were collected from the supernatant after 12,000 rpm centrifugation at 4°C for 10 min and frozen at −80°C for use.

### DNA extraction and 16S rRNA gene sequencing.

The total DNA of the gut microbiota was extracted from the fecal sample by utilizing the modified cetyl trimethyl-ammonium bromide (CTAB) methods (26). To analyze the taxonomic composition of the bacterial community, the V1 to V2 region of the 16S rRNA gene was selected for the subsequent pyrosequencing. The specific primer pair 27F (5′-AGAGTTTGATCMTGGCTCAG-3′) and 355R (5′-GCTGCCTCCCGTAGGAGT-3′) were used for the PCR amplification. The 8 bp Barcode sequence and 5 to 8 random sequences were introduced into the 5′ ends of the primers to distinguish samples and remove chimeric sequences and PCR redundancy. The amplification of each sample was performed in triplicate, then products were purified and quantified, and sequenced by Illumina HiSeq 2500 sequencing platform.

### Raw data processing and analysis.

Raw sequencing data were processed and analyzed using Quantitative Insights into Microbial Ecology 2 (QIIME2, version2020.2) (27). Briefly, paired-end reads were assigned to samples by barcodes and then the barcodes and primer sequence were cut off. Next, we used the q2-dada2 (28) plugin in QIIME2 for quality control, chimera detection and deletion, and generation of amplicon sequence variants (ASVs) and representative sequences. The SILVA database (version 138) (29) classifier was used to annotate the ASVs with 99% similarity. Samples with <10 ASV features and <10,000 reads, and representative sequences with a frequency of <10 were removed. Finally, a total of 1,293 ASVs and 73 samples (35 insomnia patients and 38 healthy participants) were retained, with the number of reads ranging from 17,165 to 100,153.

We compared alpha diversity, beta diversity, and taxa between insomnia and healthy controls as follows. Alpha diversity was measured with the Observed ASVs, Shannon, Simpson, and Chao1 indexes using the get_alphaindex function in the MicrobiotaProcess R package (version 1.2.0) (30). Beta diversity was measured as the Bray-Curtis distance using the vegdist function in vegan R package (version 2.5-7) (31) after normalization by Hellinger transformation. Differences between the two groups were then compared using the Wilcoxon rank sum test for alpha diversity and the permutational multivariate analysis of variance (PERMANOVA) for beta diversity. Linear discriminant analysis (LDA) effect size (LEfSe) analysis (LDA > 2) and random forest (the intersection of the top 20 genera with the highest accuracy and the top 20 with the highest Gini index) were conducted to identify representative genera and species for the two groups using the diff_analysis function in MicrobiotaProcess R package and the randomForest function in randomForest R package (version 4.6-14) (32) with default parameters, respectively. The co-occurrence networks in the microbial communities for insomnia and healthy controls were inferred from the neighborhood algorithm, Meinshausen-Bühlmann (MB) method using SpiecEasi R package (version 1.1.2) (33) with default parameters at the genus level.

### Serum metabolites analysis based on UHPLC-MS/MS.

The 100 μL serum sample was thawed on the ice, and then 300 μL methanol (containing the internal standard, L-2-chlorophenyl alanine 1 μg/mL) was added into the tube and mixed by a vortex mixer for 30 s. After the ultrasonic treatment in the ice-water bath for 10 min, the protein was precipitated at −20°C for 1 h. The samples were centrifuged at 4°C for 15 min at 12,000 rpm. For each sample, 2 μL of the supernatant was used for the UHPLC-MS/MS analysis.

We performed chromatographic analysis on a UHPLC system (1290, Agilent Technologies) equipped with an HSS T3 column (2.1 mm × 100 mm, 1.8 μm) coupled to Q Exactive (Orbitrap MS, Thermo). The mobile phase composition was as follows: (i) positive ion mode, mobile phase A, 0.1% formic acid aqueous solution; mobile phase B, acetonitrile; (ii) anion mode, mobile phase A, 5 mmol/L ammonium acetate aqueous solution; mobile phase B, acetonitrile.

The first and second mass spectrometry data were collected by the Thermo Q Exactive Orbitrap mass spectrometer under the Xcalibur software (version: 4.0.27, Thermo). Bombardment energy (normalized collision energy mode) was 20 eV, 40 eV, or 60 eV, and the scanning rate was 7 Hz.

ProteoWizard software was used to convert the original mass spectrometry data into mzML format. The retention time, identify peaks, extract peaks, integrate peaks, and align peaks were corrected by XCMS (version 3.2). Material identification was carried out by using OSI-SMMS (version 1.0) software and a self-built database. The log conversion and par formatting of data were carried out by using soft independent modelling of class analogy (SIMCA, version 14.1). The normalized data were then to perform the principal component analysis (PCA) and orthogonal projections to latent structures-discriminant analysis (OPLS-DA). The metabolites were screened by the variable importance in the project (VIP) >1 in the OPLS-DA model (34). The screened metabolites were then mapped to the KEGG, PubChem, and HMDB databases. Metabo Analyst software (http://www.metaboanalyst.ca/) was used for metabolic pathway analysis (35). The Student's *t* test (*P* < 0.05) was further used to identify differential metabolites.

### Detection of the serum immune factors.

The levels of the immune factors were measured by a human IL-1β/IL-6/TNF-α ELISA kit (ABclonal Technology) with 100 μL serum in each test according to the procedures supplied by the manufacturer. The absorbance of each sample was measured by Multiplate Reader Ascent (Thermo) at 450 nm and 630 nm, and the amount of protein in pg/mL was calculated based on a standard curve. Each serum sample was tested in triplicate, and the precision and accuracy of the assay as determined by intraassay coefficient variation was below 10%.

### Multiomics analyses.

Correlation analysis of immune factors, differential metabolites, and signature microbes based on Spearman rank correlation coefficient was done with the R packages Hmisc (version 4.6.0) (34) and corrplot (version 0.90) (35). We used multivariate analyses to explore the interrelationships between the microbes and immune factors/metabolites. First, the microbial community distance matrix was calculated based on Bray-Curtis distance using the R package vegan. Then, metabolites relevant to the microbial community were filtered using PERMANOVA for subsequent multivariate analyses. Furthermore, Metabolites with variance inflation factor (VIF) >10 were excluded to reduce multiple colinear relationships. Only the genus or species identified by both LEfSe and random forest were retained for subsequent redundancy analysis (RDA). Next, we applied RDA to assess how much of the variation in selected microbes can be explained by the variation in selected metabolites and immune factors using the R package vegan.

Additionally, we performed mediation analysis using the mediation R package (version 4.5.0) (36) to explore the underlying causal pathway among gut microbiota, inflammatory factors, metabolites, and the development of insomnia. Representative microbes identified by LEfSe and random forest, inflammatory factors, and differential metabolites were included in the mediation analysis. The species and genus count data were transformed to log counts per million + 1 (log[CPM + 1]). Then, we applied mediation analysis to the gut microbiome-inflammatory factors/metabolites-insomnia triplet. The metabolites and inflammatory factors were used as candidate mediators. Lastly, the gut microbes were used as candidate mediators to explore the inflammatory factors/metabolites-gut microbiome-insomnia axis.

### Statistical analyses.

All statistical analyses and graphs were done with R software (version 4.0.3). Data were presented as mean ± SD. Statistical comparisons were analyzed by the Wilcoxon rank sum test and Chi-squared tests for categorical and continuous variables, respectively. The false discovery rate (FDR) was used to correct multiple testing. *P* < 0.05 was statistically significant.

### Data availability.

All raw sequencing data created in this study have been uploaded to the National Omics Data Encyclopedia (NODE; https://www.biosino.org/node/index) with the accession number OEP002524.
