# Supplementary material for: Multiomics Analysis Reveals Aberrant Metabolism and Immunity Linked Gut Microbiota with Insomnia
Source: Microbiol Spectr. 2022 Oct 3;10(5):e00998-22. doi: 10.1128/spectrum.00998-22 (PMC9602994; doi:10.1128/spectrum.00998-22)
Supplement: Supplemental file 1 — Supplemental material. Download spectrum.00998-22-s0001.pdf, PDF file, 0.9 MB [file spectrum.00998-22-s0001.pdf]

Supplementary materials for the manuscript:

Multi-Omics analysis reveals aberrant metabolism and immunity linked gut microbiota with insomnia

Qinghua Wang, Bin Chen, Dashuang Sheng, Junjie Yang, Shujie Fu, Jingwen Wang, Changying Zhao, Yihui Wang, Xiangzhen Gai, Jianfeng Wang, Kyle Stirling, Xueyuan Heng, Honghao Man, Lei Zhang

This file includes:

Figures S1-S6

Table S1-S3

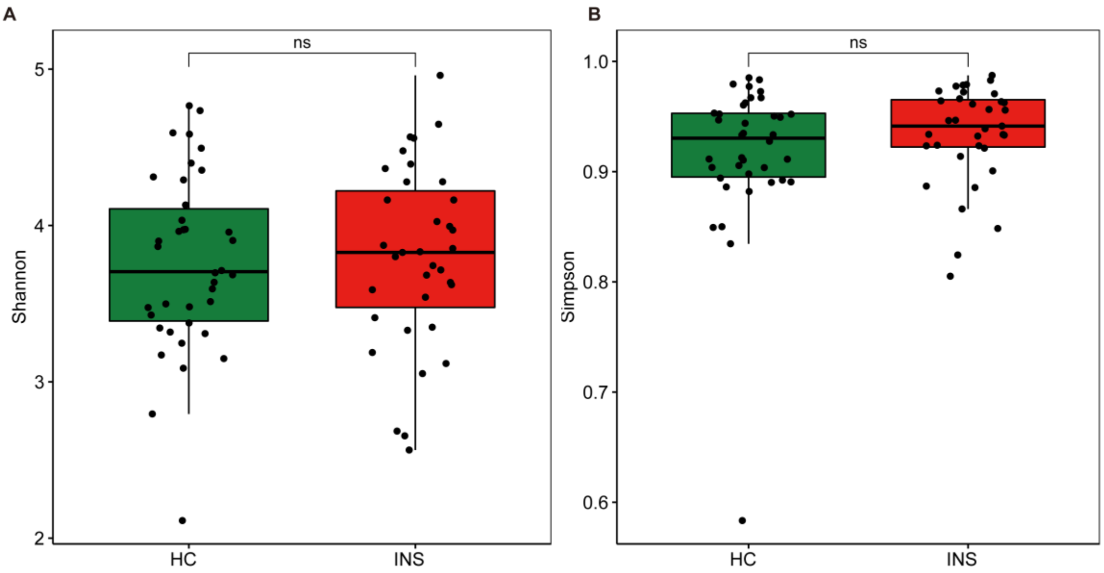

Figure S1 Alpha diversity of gut microbiota in INS and HC.
(A) Shannon index. **(B)** Simpson index.

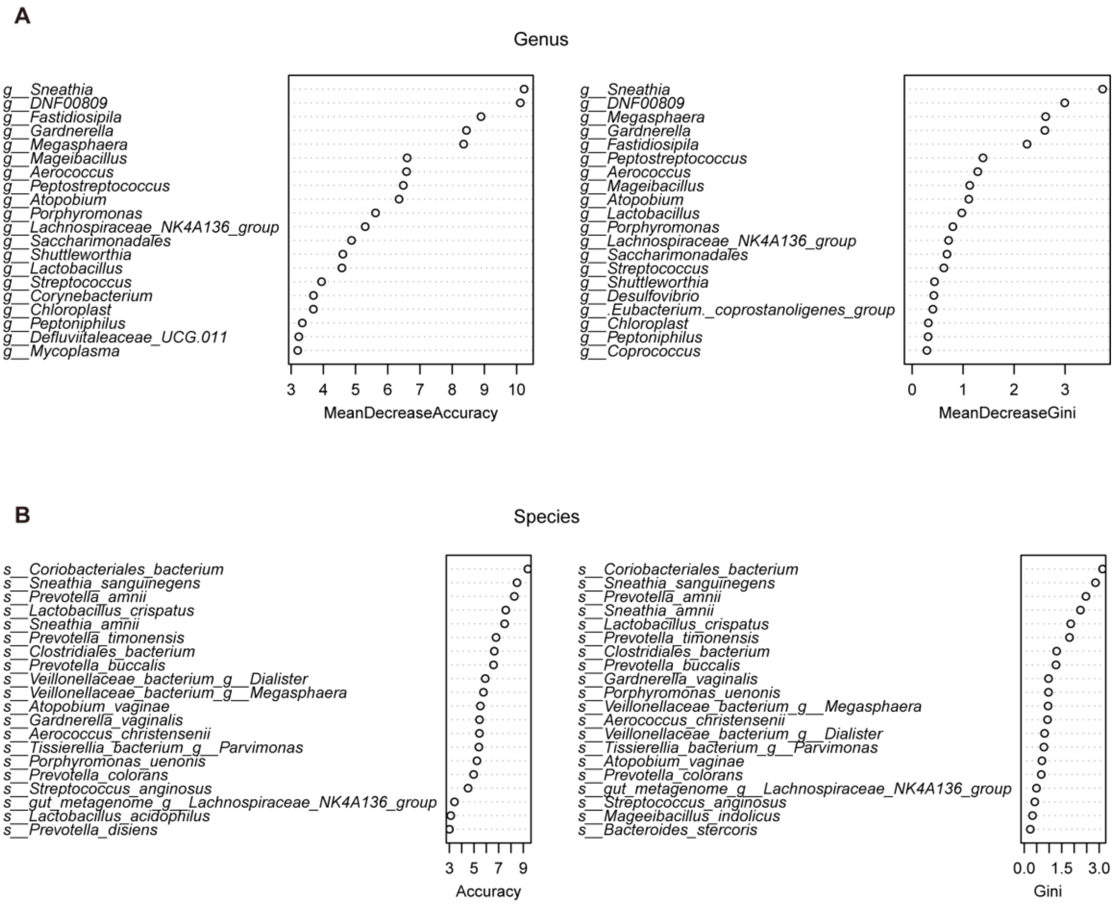

Figure S2 Microbial features identified by random forest.
(A) Genus level. **(B)** Species level.

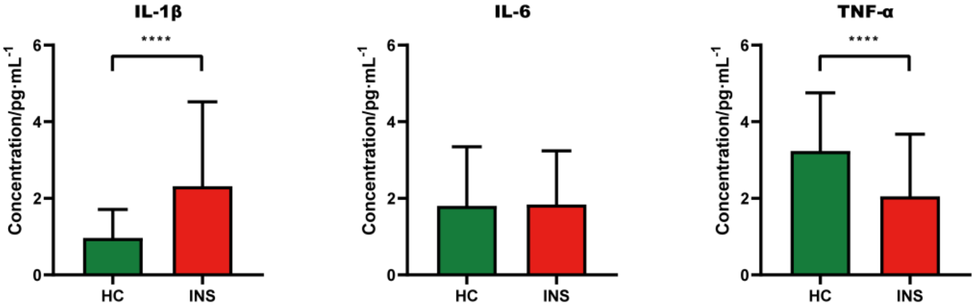

Figure S3 Inflammatory factors level in serum as assessed by ELISA.

(A-C) Comparisons of IL-1β **(A)**, IL-6 **(B)** and TNF-α **(C)** level between insomniacs and healthy controls. (*** $P < 0.001$, **** $P < 0.0001$)

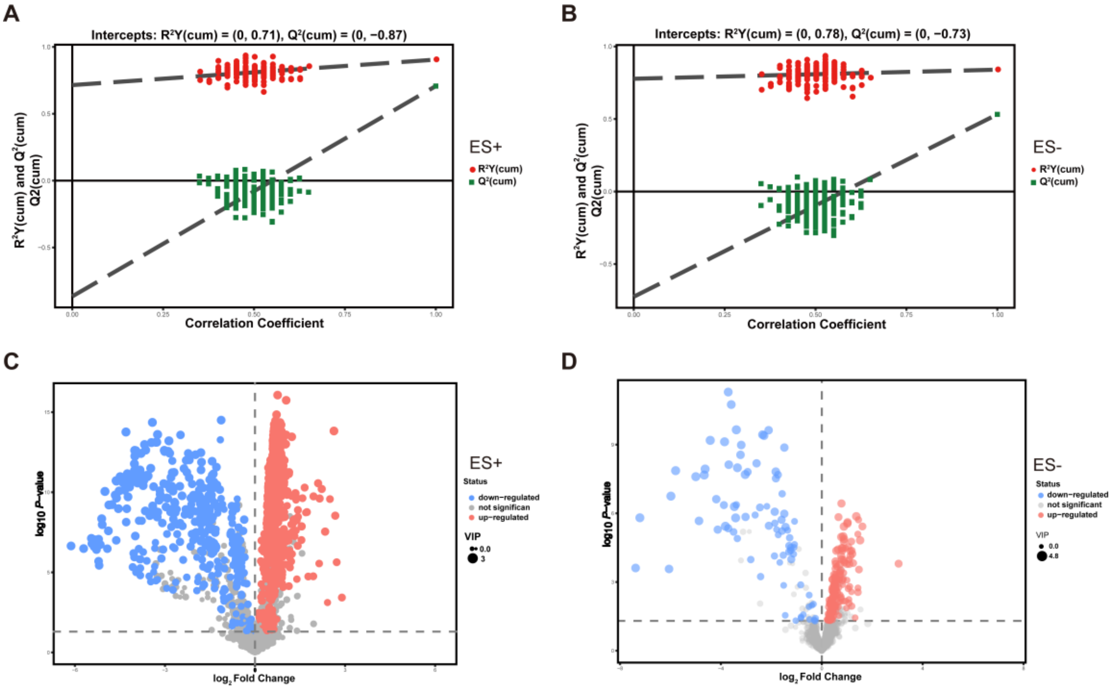

Figure S4 Differential metabolite between insomniacs and healthy controls.
(A) Permutation test of OPLS-DA model in positive ion mode. **(B)** Permutation test of OPLS-DA model in negative ion mode. **(C)** Volcano plot for differential metabolites in positive ion mode. **(D)** Volcano plot for differential metabolites in negative ion mode.

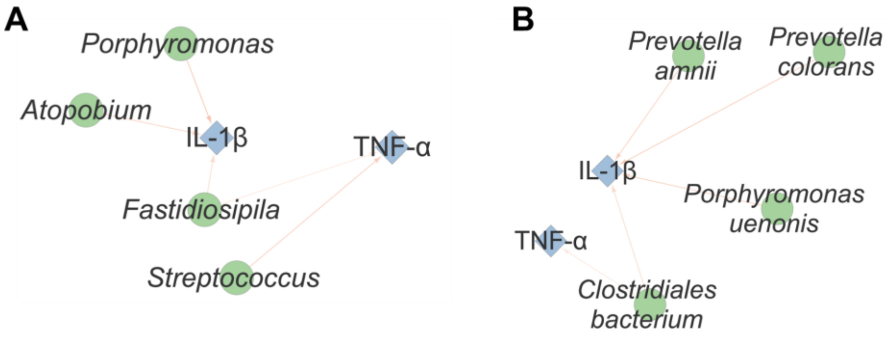

Figure S5 Inflammatory factors mediate the effect of gut microbes on insomnia
(A) Genus level. **(B)** Species level. Green and violet indicate signature microbes and Inflammatory factors, respectively.

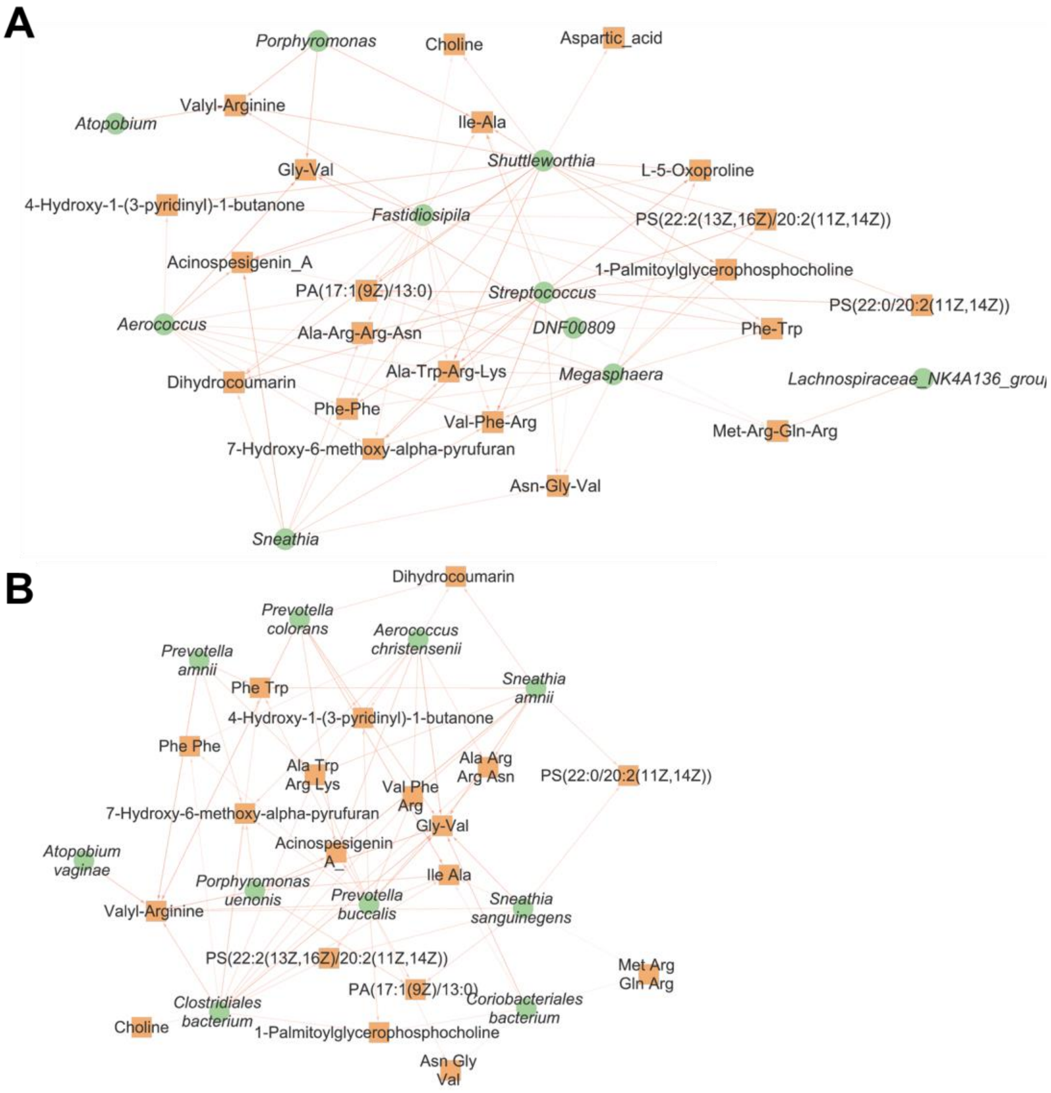

Figure S6 Metabolites mediate the effect of gut microbes on insomnia
(A) Genus level. **(B)** Species level. Green and brown indicate signature microbes and metabolites, respectively.

Table S1 Characteristics of participants

	Insomnia	Healthy control	P value
Number	40	40	/
sexuality（male: female）	13:27	10:30	0.459
Average age（years）	59.23±9.35	55.85±9.02	0.0534
Age distribution（years）	40~77	43~74	/
BMI(kg/m^2)	25.08±3.55	23.81±1.35	0.3293

Table S2 Differential metabolites between INS and HC

	Metabolite	Mean HC	Mean INS	VIP	P	FDR P	LOG foldchange
ES+	Choline	0.0189	0.0148	1.3785	0.0000	0.0001	0.3552
	Ile Ala	0.0077	0.0189	1.4112	0.0000	0.0000	-1.3016
	Phenyl beta-D-glucopyranoside	0.0094	0.0070	1.4602	0.0051	0.0129	0.4275
	Phe Ile	0.0480	0.0337	1.3005	0.0080	0.0197	0.5104
	7-Hydroxy-6-methoxy-alpha-pyrufuran	0.0035	0.0045	2.3572	0.0000	0.0000	-0.3606
	Asp-Phe	0.0541	0.0374	1.2944	0.0071	0.0178	0.5331
	Gly-Val	0.0620	0.0100	2.6489	0.0000	0.0000	2.6292
	Acinospesigenin A	0.0073	0.0191	2.5864	0.0000	0.0000	-1.3778
	Dihydrocoumarin	0.0665	0.0542	1.6435	0.0000	0.0000	0.2954
	Valyl-Arginine	0.0022	0.0079	2.0499	0.0000	0.0000	-1.8374
	Asn Gly Val	0.0163	0.0071	1.6236	0.0000	0.0000	1.2044
	4-Hydroxy-1-(3-pyridinyl)-1-butanone	3.9125	3.1585	1.8050	0.0000	0.0000	0.3088
	Met Arg Gln Arg	0.0525	0.0392	1.3235	0.0001	0.0002	0.4226
	PS(22:2(13Z,16Z)/20:2(11Z,14Z))	0.0018	0.0053	2.1353	0.0000	0.0000	-1.5356
	PA(17:1(9Z)/13:0)	0.0096	0.0216	2.3691	0.0000	0.0000	-1.1709
	L-5-Oxoproline	0.2732	0.2028	1.9814	0.0000	0.0000	0.4296
	Indoline	0.0068	0.0048	1.1596	0.0024	0.0064	0.5237
	Gamma-Glutamylglutamine	0.0357	0.0405	1.1760	0.0101	0.0245	-0.1831
	1-Palmitoylglycerophosphocholine	6.8267	4.7839	2.0074	0.0000	0.0000	0.5130
	PS(22:0/20:2(11Z,14Z))	0.0022	0.0057	1.6778	0.0000	0.0000	-1.3815
	Phe Phe	0.7016	0.4329	1.7200	0.0000	0.0000	0.6967
	Ala Arg Arg Asn	0.0162	0.0121	2.1370	0.0000	0.0000	0.4258
	Phe Trp	0.0363	0.0229	1.9968	0.0000	0.0000	0.6630
	Val Phe Arg	0.0834	0.0609	2.2072	0.0000	0.0000	0.4532
	Ala Trp Arg Lys	0.0265	0.0194	2.1931	0.0000	0.0000	0.4518
ES-	Aspartic acid	11.5419	6.3998	3.3054	0.0000	0.0004	0.8508
	Phenylalanine	6.7527	5.2684	2.0338	0.0058	0.0628	0.3581
	Threonic acid	10.9590	8.2697	1.9981	0.0018	0.0252	0.4062
	1-Palmitoyl Lysophosphatidic Acid	0.5500	0.4426	2.1048	0.0226	0.1788	0.3133
	cholesterol sulfate	0.3101	0.4071	1.0680	0.0105	0.0992	-0.3928
	Phosphatidylcholine lyso 20:4	4.4194	3.0724	2.5102	0.0013	0.0199	0.5245

Table S3 Metabolites and inflammatory factors mediate gut microbiota and insomnia

**Bacterial taxon**	**mediator**	**Prop of Mediated Estimation**	**Prop of Mediated P**	**Prop of Mediated FDR P**
Streptococcus	Acinospesigenin A	0.6728	0.0000	0.0000
Streptococcus	Gly-Val	0.5912	0.0000	0.0000
Streptococcus	Val Phe Arg	0.5244	0.0000	0.0000
Streptococcus	L-5-Oxoproline	0.5108	0.0000	0.0000
Streptococcus	PS(22:2(13Z,16Z)/20:2(11Z,14Z))	0.5098	0.0000	0.0000
Streptococcus	Ala Trp Arg Lys	0.4703	0.0000	0.0000
Streptococcus	PA(17:1(9Z)/13:0)	0.4701	0.0000	0.0000
Streptococcus	Dihydrocoumarin	0.4686	0.0000	0.0000
Streptococcus	Ala Arg Arg Asn	0.4510	0.0000	0.0000
Streptococcus	PS(22:0/20:2(11Z,14Z))	0.4436	0.0000	0.0000
Streptococcus	7-Hydroxy-6-methoxy-alpha-pyrufuran	0.4433	0.0000	0.0000
Streptococcus	Phe Trp	0.3945	0.0000	0.0000
Streptococcus	Ile Ala	0.3781	0.0000	0.0000
Streptococcus	1-Palmitoylglycerophosphocholine	0.3665	0.0000	0.0000
Streptococcus	Phe Phe	0.3328	0.0000	0.0000
Streptococcus	Asn Gly Val	0.2567	0.0000	0.0000
Streptococcus	TNF.α	0.2339	0.0000	0.0000
Sneathia sanguinegens	Gly-Val	0.3900	0.0000	0.0000
Sneathia sanguinegens	Valyl-Arginine	0.2405	0.0000	0.0000
Sneathia sanguinegens	PA(17:1(9Z)/13:0)	0.2402	0.0000	0.0000
Sneathia sanguinegens	PS(22:0/20:2(11Z,14Z))	0.2139	0.0000	0.0000
Sneathia sanguinegens	PS(22:2(13Z,16Z)/20:2(11Z,14Z))	0.2074	0.0000	0.0000
Sneathia sanguinegens	Ile Ala	0.1156	0.0000	0.0000
Sneathia sanguinegens	Met Arg Gln Arg	0.0255	0.0000	0.0000
Sneathia amnii	Acinospesigenin A	0.4999	0.0000	0.0000
Sneathia amnii	Gly-Val	0.4648	0.0000	0.0000
Sneathia amnii	Phe Trp	0.3422	0.0000	0.0000
Sneathia amnii	Val Phe Arg	0.3105	0.0000	0.0000
Sneathia amnii	Ala Arg Arg Asn	0.2990	0.0000	0.0000
Sneathia amnii	Ala Trp Arg Lys	0.2870	0.0000	0.0000
Sneathia amnii	Dihydrocoumarin	0.2518	0.0000	0.0000
Sneathia amnii	PS(22:0/20:2(11Z,14Z))	0.2242	0.0000	0.0000
Sneathia	Acinospesigenin A	0.4998	0.0000	0.0000
Sneathia	Val Phe Arg	0.3150	0.0000	0.0000
Sneathia	Ala Arg Arg Asn	0.3007	0.0000	0.0000
Sneathia	Ala Trp Arg Lys	0.2901	0.0000	0.0000
Sneathia	Dihydrocoumarin	0.2513	0.0200	0.0497
Sneathia	Phe Phe	0.2219	0.0000	0.0000
Sneathia	Asn Gly Val	0.1950	0.0200	0.0497

Shuttleworthia	Gly-Val	0.5881	0.0200	0.0497
Shuttleworthia	Ala Arg Arg Asn	0.5628	0.0000	0.0000
Shuttleworthia	Val Phe Arg	0.5366	0.0000	0.0000
Shuttleworthia	Ala Trp Arg Lys	0.5225	0.0000	0.0000
Shuttleworthia	Acinospesigenin A	0.4879	0.0000	0.0000
Shuttleworthia	PA(17:1(9Z)/13:0)	0.4655	0.0000	0.0000
Shuttleworthia	1-Palmitoylglycerophosphocholine	0.4629	0.0000	0.0000
Shuttleworthia	Valyl-Arginine	0.4499	0.0000	0.0000
Shuttleworthia	4-Hydroxy-1-(3-pyridinyl)-1-butanone	0.4092	0.0000	0.0000
Shuttleworthia	Ile Ala	0.4072	0.0000	0.0000
Shuttleworthia	L-5-Oxoproline	0.3904	0.0000	0.0000
Shuttleworthia	Dihydrocoumarin	0.3807	0.0000	0.0000
Shuttleworthia	PS(22:2(13Z,16Z)/20:2(11Z,14Z))	0.3567	0.0000	0.0000
Shuttleworthia	PS(22:0/20:2(11Z,14Z))	0.3061	0.0000	0.0000
Shuttleworthia	Phe Phe	0.3047	0.0000	0.0000
Shuttleworthia	Phe Trp	0.2932	0.0000	0.0000
Shuttleworthia	Choline	0.2216	0.0200	0.0497
Shuttleworthia	Aspartic acid	0.1982	0.0200	0.0497
Prevotella colorans	Valyl-Arginine	0.4733	0.0000	0.0000
Prevotella colorans	Ile Ala	0.4212	0.0000	0.0000
Prevotella colorans	Gly-Val	0.3816	0.0000	0.0000
Prevotella colorans	Acinospesigenin A	0.3225	0.0000	0.0000
Prevotella colorans	IL.1β	0.2533	0.0000	0.0000
Prevotella colorans	4-Hydroxy-1-(3-pyridinyl)-1-butanone	0.2515	0.0000	0.0000
Prevotella colorans	Dihydrocoumarin	0.2271	0.0000	0.0000
Prevotella buccalis	Gly-Val	0.4698	0.0000	0.0000
Prevotella buccalis	Acinospesigenin A	0.3266	0.0000	0.0000
Prevotella buccalis	Val Phe Arg	0.3040	0.0000	0.0000
Prevotella buccalis	Valyl-Arginine	0.2810	0.0000	0.0000
Prevotella buccalis	Ala Arg Arg Asn	0.2692	0.0000	0.0000
Prevotella buccalis	PA(17:1(9Z)/13:0)	0.2664	0.0000	0.0000
Prevotella buccalis	Ala Trp Arg Lys	0.2627	0.0000	0.0000
Prevotella buccalis	1-Palmitoylglycerophosphocholine	0.2612	0.0000	0.0000
Prevotella buccalis	Ile Ala	0.2453	0.0000	0.0000
Prevotella buccalis	4-Hydroxy-1-(3-pyridinyl)-1-butanone	0.2349	0.0000	0.0000
Prevotella buccalis	Phe Trp	0.2346	0.0000	0.0000
Prevotella buccalis	PS(22:2(13Z,16Z)/20:2(11Z,14Z))	0.2251	0.0000	0.0000
Prevotella buccalis	7-Hydroxy-6-methoxy-alpha-pyrufuran	0.2069	0.0000	0.0000
Prevotella buccalis	Asn Gly Val	0.1374	0.0000	0.0000
Prevotella buccalis	Phe Phe	0.1298	0.0000	0.0000
Prevotella amnii	Valyl-Arginine	0.4992	0.0000	0.0000
Prevotella amnii	Phe Trp	0.2867	0.0000	0.0000
Prevotella amnii	Ala Trp Arg Lys	0.2740	0.0000	0.0000
Prevotella amnii	7-Hydroxy-6-methoxy-alpha-pyrufuran	0.2563	0.0000	0.0000

Prevotella amnii	IL.1β	0.2282	0.0000	0.0000
Porphyromonas uenonis	Gly-Val	0.5302	0.0000	0.0000
Porphyromonas uenonis	Valyl-Arginine	0.4786	0.0000	0.0000
Porphyromonas uenonis	Acinospesigenin A	0.3945	0.0000	0.0000
Porphyromonas uenonis	Ile Ala	0.3747	0.0000	0.0000
Porphyromonas uenonis	PA(17:1(9Z)/13:0)	0.3070	0.0000	0.0000
Porphyromonas uenonis	7-Hydroxy-6-methoxy-alpha-pyrufuran	0.2508	0.0000	0.0000
Porphyromonas uenonis	IL.1β	0.2308	0.0000	0.0000
Porphyromonas	Valyl-Arginine	0.4711	0.0000	0.0000
Porphyromonas	Gly-Val	0.4437	0.0000	0.0000
Porphyromonas	Ile Ala	0.4335	0.0000	0.0000
Porphyromonas	IL.1β	0.2940	0.0000	0.0000
Megasphaera	Val Phe Arg	0.3369	0.0000	0.0000
Megasphaera	PA(17:1(9Z)/13:0)	0.3191	0.0000	0.0000
Megasphaera	1-Palmitoylglycerophosphocholine	0.3145	0.0000	0.0000
Megasphaera	Ala Arg Arg Asn	0.2978	0.0000	0.0000
Megasphaera	Ala Trp Arg Lys	0.2886	0.0000	0.0000
Megasphaera	PS(22:2(13Z,16Z)/20:2(11Z,14Z))	0.2721	0.0000	0.0000
Megasphaera	L-5-Oxoproline	0.2709	0.0200	0.0497
Megasphaera	Phe Trp	0.2642	0.0000	0.0000
Megasphaera	7-Hydroxy-6-methoxy-alpha-pyrufuran	0.2498	0.0000	0.0000
Megasphaera	Phe Phe	0.2241	0.0000	0.0000
Megasphaera	Asn Gly Val	0.1933	0.0000	0.0000
Megasphaera	Met Arg Gln Arg	0.1156	0.0000	0.0000
Lachnospiraceae_NK4A136_group	Phe Trp	0.7473	0.0000	0.0000
Lachnospiraceae_NK4A136_group	PS(22:0/20:2(11Z,14Z))	0.6132	0.0000	0.0000
Lachnospiraceae_NK4A136_group	Met Arg Gln Arg	0.2763	0.0200	0.0497
Fastidiosipila	Gly-Val	0.4507	0.0000	0.0000
Fastidiosipila	Valyl-Arginine	0.3504	0.0000	0.0000
Fastidiosipila	Acinospesigenin A	0.2620	0.0000	0.0000
Fastidiosipila	L-5-Oxoproline	0.2356	0.0200	0.0497
Fastidiosipila	Val Phe Arg	0.2231	0.0000	0.0000
Fastidiosipila	Ile Ala	0.2179	0.0000	0.0000
Fastidiosipila	Ala Trp Arg Lys	0.2134	0.0000	0.0000
Fastidiosipila	Phe Trp	0.2118	0.0000	0.0000
Fastidiosipila	7-Hydroxy-6-methoxy-alpha-pyrufuran	0.2099	0.0000	0.0000
Fastidiosipila	PA(17:1(9Z)/13:0)	0.2093	0.0200	0.0497
Fastidiosipila	Ala Arg Arg Asn	0.1956	0.0000	0.0000
Fastidiosipila	1-Palmitoylglycerophosphocholine	0.1867	0.0000	0.0000
Fastidiosipila	4-Hydroxy-1-(3-pyridinyl)-1-butanone	0.1867	0.0200	0.0497
Fastidiosipila	PS(22:2(13Z,16Z)/20:2(11Z,14Z))	0.1758	0.0000	0.0000

Fastidiosipila	Dihydrocoumarin	0.1667	0.0200	0.0497
Fastidiosipila	IL.1β	0.1511	0.0000	0.0000
Fastidiosipila	Phe Phe	0.1291	0.0000	0.0000
Fastidiosipila	Choline	0.0997	0.0000	0.0000
Fastidiosipila	TNF.α	0.0577	0.0000	0.0000
DNF00809	Gly-Val	0.4201	0.0000	0.0000
DNF00809	1-Palmitoylglycerophosphocholine	0.1301	0.0000	0.0000
DNF00809	Acinospesigenin A	0.1282	0.0200	0.0497
DNF00809	Val Phe Arg	0.1217	0.0200	0.0497
DNF00809	Phe Trp	0.1199	0.0200	0.0497
DNF00809	Ala Trp Arg Lys	0.1096	0.0200	0.0497
DNF00809	Ala Arg Arg Asn	0.1071	0.0200	0.0497
DNF00809	PA(17:1(9Z)/13:0)	0.1043	0.0000	0.0000
DNF00809	Ile Ala	0.1011	0.0000	0.0000
DNF00809	Asn Gly Val	0.0589	0.0000	0.0000
DNF00809	Met Arg Gln Arg	0.0465	0.0000	0.0000
Coriobacteriales bacterium	Gly-Val	0.4201	0.0000	0.0000
Coriobacteriales bacterium	1-Palmitoylglycerophosphocholine	0.1301	0.0000	0.0000
Coriobacteriales bacterium	PA(17:1(9Z)/13:0)	0.1043	0.0000	0.0000
Coriobacteriales bacterium	Ile Ala	0.1011	0.0000	0.0000
Coriobacteriales bacterium	Asn Gly Val	0.0589	0.0000	0.0000
Coriobacteriales bacterium	Met Arg Gln Arg	0.0465	0.0000	0.0000
Clostridiales bacterium	Gly-Val	0.4507	0.0000	0.0000
Clostridiales bacterium	Valyl-Arginine	0.3504	0.0000	0.0000
Clostridiales bacterium	Acinospesigenin A	0.2620	0.0000	0.0000
Clostridiales bacterium	Val Phe Arg	0.2231	0.0000	0.0000
Clostridiales bacterium	Ile Ala	0.2179	0.0000	0.0000
Clostridiales bacterium	Ala Trp Arg Lys	0.2134	0.0000	0.0000
Clostridiales bacterium	Phe Trp	0.2118	0.0000	0.0000
Clostridiales bacterium	7-Hydroxy-6-methoxy-alpha-pyrufuran	0.2099	0.0000	0.0000
Clostridiales bacterium	Ala Arg Arg Asn	0.1956	0.0000	0.0000
Clostridiales bacterium	1-Palmitoylglycerophosphocholine	0.1867	0.0000	0.0000
Clostridiales bacterium	PS(22:2(13Z,16Z)/20:2(11Z,14Z))	0.1758	0.0000	0.0000
Clostridiales bacterium	IL.1β	0.1511	0.0000	0.0000
Clostridiales bacterium	Phe Phe	0.1291	0.0000	0.0000
Clostridiales bacterium	Choline	0.0997	0.0000	0.0000
Clostridiales bacterium	TNF.α	0.0577	0.0000	0.0000
Atopobium vaginae	Valyl-Arginine	0.4529	0.0000	0.0000

Atopobium	Valyl-Arginine	0.4912	0.0000	0.0000
Atopobium	IL.1β	0.2011	0.0200	0.0497
Aerococcus christensenii	Gly-Val	0.4895	0.0000	0.0000
Aerococcus christensenii	Acinospesigenin A	0.3547	0.0000	0.0000
Aerococcus christensenii	7-Hydroxy-6-methoxy-alpha-pyrufuran	0.2522	0.0000	0.0000
Aerococcus christensenii	Ala Trp Arg Lys	0.2466	0.0000	0.0000
Aerococcus christensenii	Val Phe Arg	0.2424	0.0000	0.0000
Aerococcus christensenii	Ala Arg Arg Asn	0.2150	0.0000	0.0000
Aerococcus christensenii	4-Hydroxy-1-(3-pyridinyl)-1-butanone	0.1918	0.0000	0.0000
Aerococcus christensenii	Dihydrocoumarin	0.1594	0.0000	0.0000
Aerococcus christensenii	Phe Phe	0.1382	0.0000	0.0000
Aerococcus	Gly-Val	0.4895	0.0000	0.0000
Aerococcus	Acinospesigenin A	0.3547	0.0000	0.0000
Aerococcus	7-Hydroxy-6-methoxy-alpha-pyrufuran	0.2522	0.0000	0.0000
Aerococcus	Ala Trp Arg Lys	0.2466	0.0000	0.0000
Aerococcus	Val Phe Arg	0.2424	0.0000	0.0000
Aerococcus	PA(17:1(9Z)/13:0)	0.2170	0.0200	0.0497
Aerococcus	Ala Arg Arg Asn	0.2150	0.0000	0.0000
Aerococcus	4-Hydroxy-1-(3-pyridinyl)-1-butanone	0.1918	0.0000	0.0000
Aerococcus	Dihydrocoumarin	0.1594	0.0000	0.0000
Aerococcus	Phe Phe	0.1382	0.0000	0.0000
